# FloodIMG: Flood image DataBase system

**DOI:** 10.1016/j.dib.2023.109164

**Published:** 2023-04-18

**Authors:** R. Karanjit, R. Pally, S. Samadi

**Affiliations:** a School of Computing, Clemson University, Clemson, SC, USA; b Agricultural Sciences Department, Clemson University, Clemson, SC, USA

**Keywords:** Application programming interface, FloodIMG, Flood image segmentation, Label detection

## Abstract

A breakthrough in building models for image processing came with the discovery that a convolutional neural network (CNN) can progressively extract higher-level representations of the image content. Having high-resolution images to train CNN models is a key for optimizing the performance of image segmentation models. This paper presents a new dataset—called Flood Image (FloodIMG) database system—that was developed for flood related image processing and segmentation. We developed various Internet of Things Application Programming Interfaces (IoT API) to gather flood-related images from Twitter, and US federal agencies’ web servers, such as the US Geological Survey (USGS) and the Department of Transportation (DOT). Overall, >9200 images of flooding events were collected, preprocessed, and formatted to make the dataset applicable for CNN training. Bounding boxes and polygon primitives were also labeled on each image to localize and classify an object in the image. Two use cases of FloodIMG are presented in this paper, where the Fast Region-based CNN (R-CNN) algorithm was used to estimate flood severity and depth during recent flooding events in the US. As of >9200 images, 7,400 were categorized as training sets, whereas >1,800 images were used for the R-CNN testing. Users can access the FloodIMG database freely through Kaggle platform to create more accessible, accurate, and optimized image segmentation models. The FloodIMG workflow concludes with a visualization of colors and labels per image that can serve as a benchmark for flood image processing and segmentation.

**Specifications Table**

**Table:** 

Subject	Hydrology and Water quality, Water Science and Technology, Data Science
Specific subject area	Computer Vision and Pattern Recognition, Water Data Science, Deep Learning Modeling, and Big Data Analytics
Type of data	Images
How the data were acquired	Images were collected from various sources like Twitter, the Department of Transport (DOT) 511 traffic cameras, and other online sources (i.e., Google search and GitHub).
Data format	Raw
Description of data collection	We used Twitter API through tweepy package in python to collect the tweets. A GitHub repository, namely “eu-flood-dataset” was used to collect images from GitHub. DOT provides live recordings of traffic cameras from various locations on its website. Using this live recording, the images were collected and preprocessed. Images from google were collected manually.
Data source location	Our data is not limited to a certain location. The dataset contains images from various flooding events occurred across the world.
Data accessibility	The dataset is freely available in Kaggle. The images can be accessed through Kaggle Python API so that users, even those with no coding skills, can view and extract the dataset. Repository name: Kaggle Direct URL to data: https://www.kaggle.com/datasets/hhrclemson/flooding-image-dataset DOI:https://doi.org/10.34740/KAGGLE/DS/1864149

## Value of the Data

• FloodIMG is created for emergency management research. The dataset can track increasing water levels during a storm and continuously monitor the potential impacts of flooding on nearby locations. FloodIMG is a comprehensive database system that can be used to train deep learning algorithms for image processing and segmentation research. FloodIMG can also be used by users who want to learn techniques and label detection methods on real-world data without much effort in data gathering and preprocessing.
• Image dataset is an essential component in computer vision-based research. Our dataset contains images from various flood events, which would make object detection for flood-related research straightforward. As data is the foundation of the deep learning models, FloodIMG will act as a benchmark dataset to train image-based flood early warning systems.
• Comprehensive image-based documentation of catastrophic events helps understand the risk associated with disaster and provides insights into the damage during catastrophic occurrences. This information is essential for reviewing and adapting flood prevention and protection principles, which serve as the foundation for mitigating the negative impacts of floods.
• An object detection model can be created using FloodIMG dataset for rescue operations. The object detection model can be loaded onto a closed-circuit television (CCTV), also known as video surveillance, camera that detects flooded objects, which help in real-time rescue operations. This automatic detection of flooded objects helps to efficiently conduct rescue operations with the integration of deep learning models.

## 1 Objective

The objective of this study is to develop an image database system that can be used to train deep-learning algorithms for flood emergency management and risk assessment. Several recent types of research (for example: [6,7]) are being conducted on water and images, but very few are related to flooding events. [6,7] contain data on general water bodies but do not specifically include flooding images. Other recent research uses already available datasets like the MS COCO dataset [8], which does not contain event-specific images. Our data contains images that are completely related to flooding events. These datasets were preprocessed and deployed in a Database system. Here, we introduce a newly developed FloodIMG dataset including object detection, label annotations, and scoring. The dataset was tested using several case studies of flooding events. Our goal was to develop an ideal dataset that can be used for training object detection and segmentation models. We anticipate that FloodIMG will enable the broader community of flood researchers to develop improved techniques for image-based flood monitoring and early warning systems. The database is easy to use and can be accessed via Kaggle, making it a proper choice for those just getting started to use image processing techniques for flood emergency management.

## 2 Data Description

### 2.1 Image Collection

**Fig. 1:**
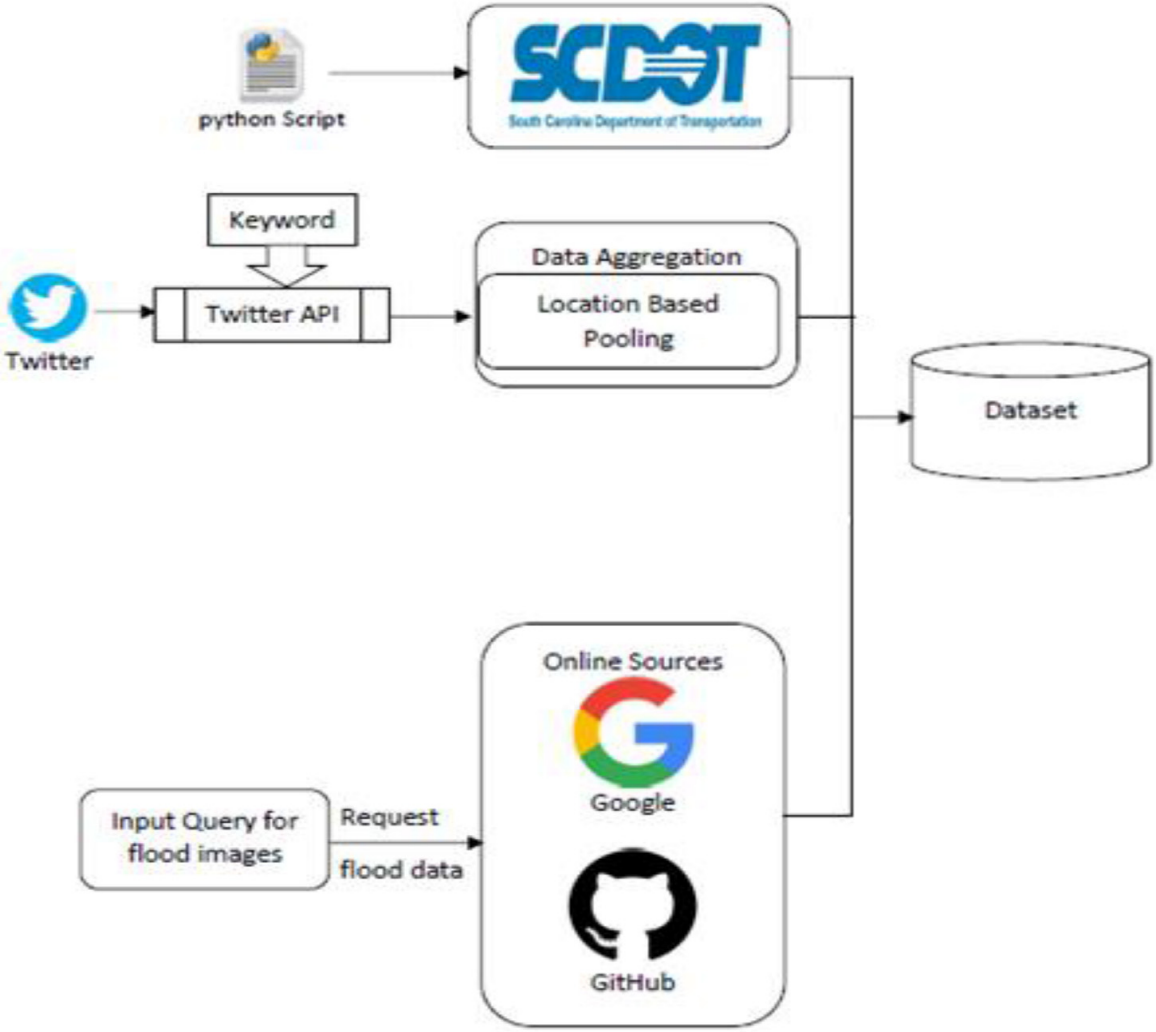
The workflow of FloodIMG database system.

**Fig. 2:**
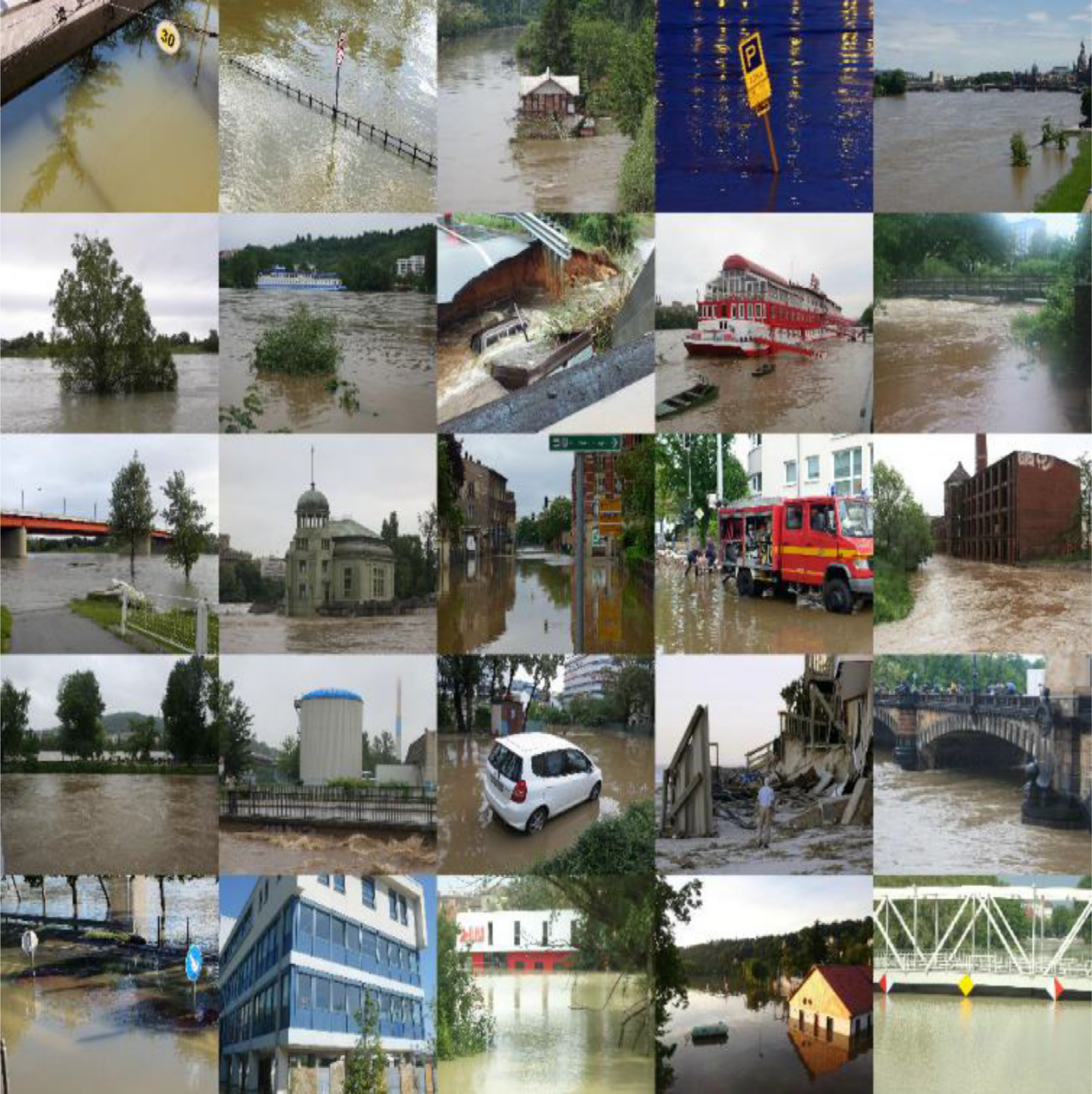
Sample of collected images.

This section discusses the sources used for data collection and the procedure used to collect images from each of the sources. In this project, we collected images from Twitter, DOT, and other online sources like Google search and GitHub [5]. We collected data from various sources so that our data will not be biased toward a specific source and we can get a variety of flood images. Various APIs were first developed to collect the data. We performed location-based pooling for Twitter data while the image meta data was used for other data sources. The images were then preprocessed and resized to the dimension of 800 × 600 that can be easily incorporated into any deep learning algorithms. Image augmentation approaches were used to rotate and zoom (flip_left_right, and flip_top_bottom functions) the images. We split the image dataset into training and test sets and annotated the images. Annotation of images involved highlighting each of the objects within an image manually using bounding boxes and labeling them appropriately. Fig. 1 illustrates the data collection architecture and workflow. Fig. 2 shows several images that are collected, resized, and preprocessed.

#### 2.1.1 Department of Transportation

DOT installed real time surveillance cameras across numerous road networks to meet the need for timely assessment of traffic and road flooding situations [3]. These real time videos/images can be processed to extract image frames and related information, which can be used to measure a range of flood characteristics such as flood depth and inundation areas. We accessed the DOT website using “urllib” python package and collected the data. The request module in “urllib” is used to open the website and then urllib.request module uses HTTP/1.1 to access the website and extract images. The exaction of images was automated with the OpenCV (Open-source Computer Vision) python library which typically takes less than a few seconds to collect the images. The live surveillance footage of DOT is cut up into individual frames, with one frame being put into the picture data repository every ten seconds. These images were then manually filtered. With the help of the OpenCV python library, frames of the video recording were extracted and stored in the local system.

#### 2.1.2 Twitter

When flooding occurs, Twitter acts as a citizen science platform for sharing flood situations and emergency information. Citizens tweet about flooding issues, damages, rescue and shelter information, and some of these tweets include images of the flood occurring in that region. The Twitter API was used to programmatically gather flood related images [11]. Real-time twitter streaming with Twitter API is quite easy, but it also has certain drawbacks. For instance, the Twitter API only allows users to view tweets from the previous seven days. We used a group of keywords such as “floods”, “flood emergency”, “disaster risk”, flooded roads”, etc., to collect Twitter images and to further filter the queries geocode i.e., the latitude and longitude values of the images that were passed to the API in order to stream geolocation information [12]. In addition, we used the latitude and longitude values of the images that were sent to the API, to stream geolocation data. Using a Twitter API through the tweepy Python module, we downloaded real-time tweets in JSON format that provided a URL for the accompanying photos to gather real-time Twitter images. We utilized the "country code" property found in the geotagged tweets JSON to filter the tweets and get photographs of flooding in the US. Collected tweets were filtered depending on whether the images contained objects like buildings, automobiles, or trees. Since only around 10% of tweets have photos linked to them [4,12], extracting and downloading photographs from Twitter required extensive time and effort.

#### 2.1.3 GitHub

A GitHub repository namely “eu-flood-dataset” contains 3,710 flood images [2]. This repository contains flood images that are related to central European floods during May to June 2013 and were retrieved from the Wikimedia Commons Category in July 2017 and its sub-categories. These images were downloaded and combined with FloodIMG dataset.

#### 2.1.4 Google

The images were collected manually from Google search engine. We searched with various keywords like floods, flood damages, flood emergency, etc. and extracted the resulting images. Overall, around 800 images of FloodIMG database system were extracted from Google search.

### 2.2 Image Data Preprocessing

We filtered all the images individually to store only those images which were related to the flood events. This results in more than 9200 images. We resized more than 9200 images to the dimension of 800 × 600. Furthermore, we separated the images collected into two parts: train and test sets. The train set contains 80% of the total images which is 7,360 images and the test set contains 20% of the total images which is >1,800 images. Separation of images into train and test is performed only to create object detection model as explained in Section 1. These images can be used for Canny Edge Detection to perform flood severity level and inundated area. Section 1 illustrates more details about these technique applications. Given an input image, the image was first resized and converted into a grayscale. Next, the skylines were identified and eliminated because both water and skyline provided the same color gradient that could be detected as a water surface. Once the skyline was eliminated, only a portion of images consisting of the water surface were included into FloodIMG.

### 2.3 Image Annotation

**Fig. 3:**
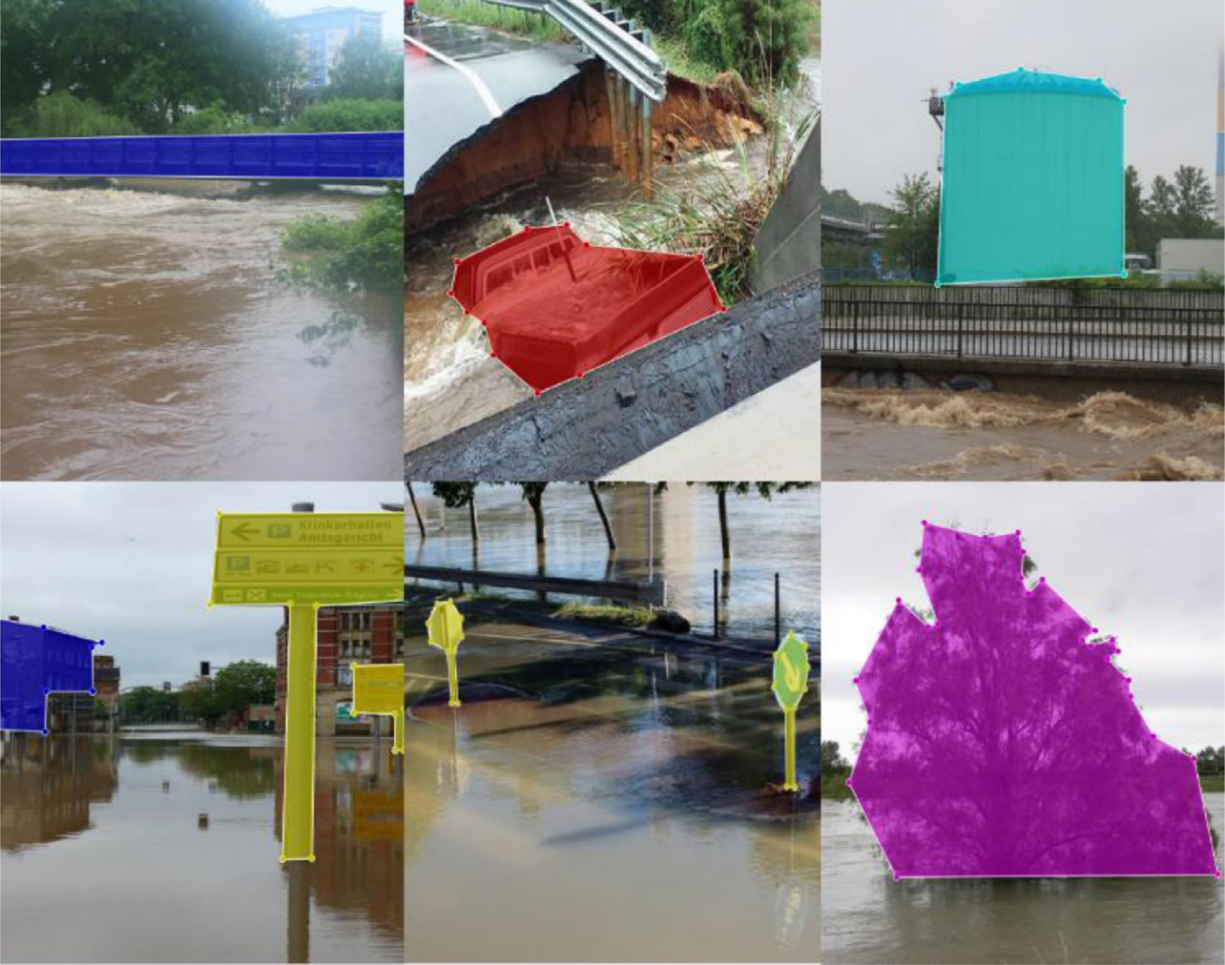
Sample of image annotation using polygon primitive.

**Fig. 4:**
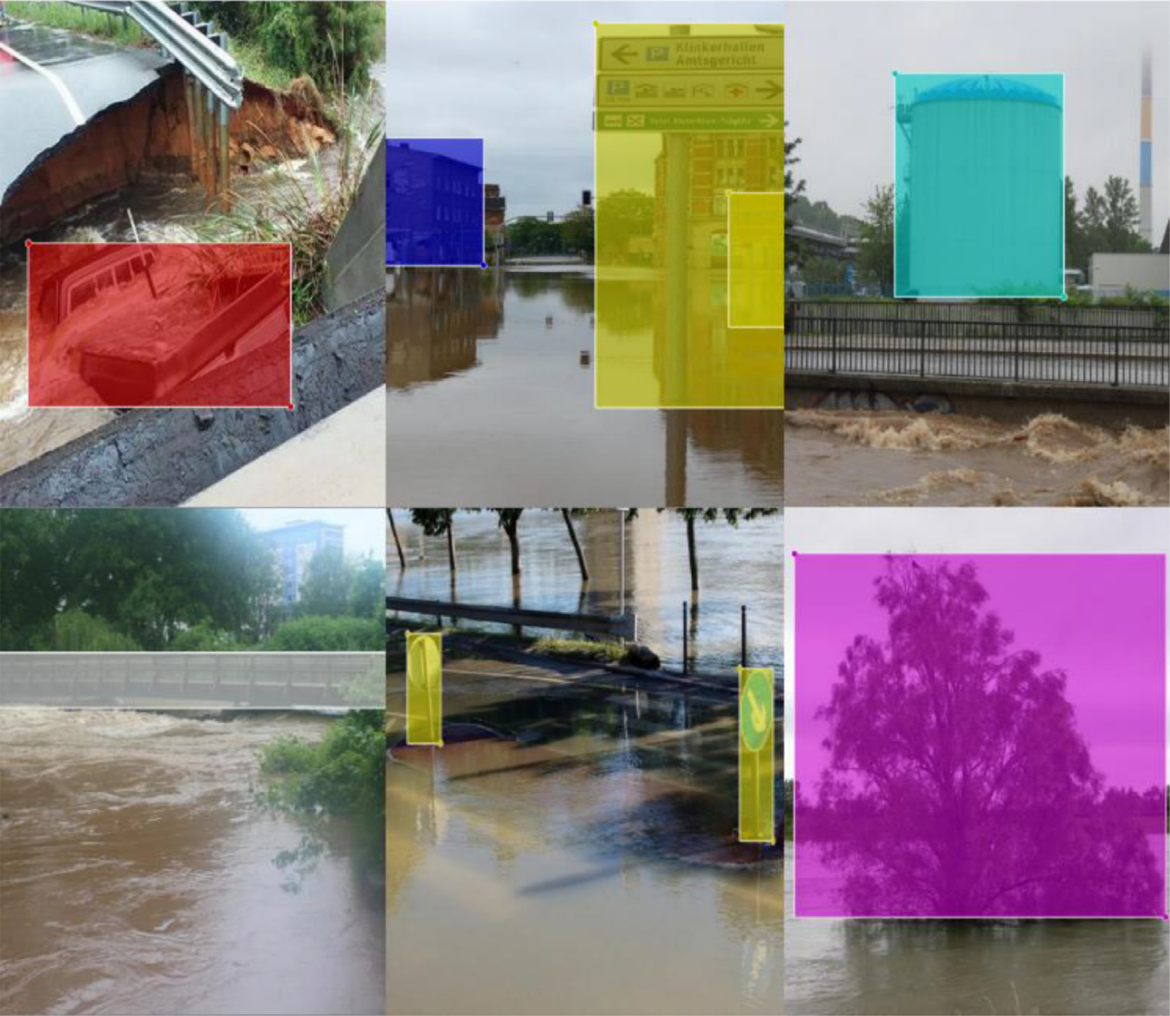
Sample of image annotation using rectangular primitive.

We annotated the images for eight objects: car, house, truck, traffic sign, tree, person, bridge, and boat. All the images were annotated using a rectangular bounding box or polygon primitive. LabelImg tool [9] was used for annotating images with a rectangular bounding box. Since LabelImg does not support annotating images with polygon primitives, we used the Labelme annotation tool for annotating images with polygon primitives. All the rectangular bounding box annotations were saved as XML files in PASCAL VOC (Visual Object Classes) format, and all the polygon annotations were saved in JSON file format. Fig. 3, Fig. 4 are the sample of image annotation which has been annotated using polygon and rectangular primitive, respectively. These annotated images can be used for object detection algorithms like fast RCNN which is illustrated in Section 1. An example of the implementation of this algorithm is also shown Section 1.

## 3 Experimental Design, Materials and Methods

FloodIMG, we believe, will become a primary resource for a wide spectrum of flood related research. One of the use cases of this dataset would be for object detection tasks. Here we provided an application of Fast R-CNN model implementation for flood label detection and probabilities of labels using FloodIMG dataset.

### 3.1 Fast Region-based CNN (R-CNN) Algorithm

Fast R-CNN is a deep CNN that is used a single, end-to-end, unified network for object detection tasks. The network can predict the positions of various items rapidly and correctly. The Fast R-CNN's design uses convolutional layers to process the entire picture and create feature maps. Then, fixed-length feature vectors are extracted from each region of interest (RoI) using pooling layers. Before being fed to the output layers, the created feature vectors are sent to the fully linked layers. Two output layers are used to create the SoftMax probabilities (a mathematical function that converts a vector of numbers into a vector of probabilities) for each category. The second output layer is also in charge of defining the bounding boxes by using four real-valued integers to represent the box's edges. The production of regional ideas is not one of the characteristics of being optimized utilizing multi-task loss. The multi-task loss function is used to jointly train classification and bounding box regression that is defined by Eq. (1).

(1) $$L(p,\;u,\;t^{u},\;v)\;=\;L_{cls}(p,\;u)\;+\;\lambda [u\;\geq \;1]L_{loc}(t^{u},\;v)\;$$

Where

• p is the output of our softmax sibling layer.
• u is ground-truth class.
• *t^u^* is output of the regressor sibling layer.
• v is bounding-box regression target.
• L_cls_ is the log loss for true class u.
• λ denotes a hyperparameter that determines the relative weight of the regression loss vs. the classification loss to the overall loss.
• [u≥1] means it is equal to 1 when u≥1.
• L_loc_ is the loss for bounding box.

$L_{cls}\left(p,\;u\right)\;=\;-\;log\;p_{u}\;$ which calculates the log loss for the classes *u* and $p_{u}$. This log loss is calculated based on the probability distribution *p = (p0, p1, … … … …., pc)* over the *C+1* output generated from the FC layer. *L_loc_(t^u^, v)* is defined over the predicted offsets $t^{u}$= ($t^{u}$x, $t^{u}$y, $t^{u}$w, $t^{u}$h) and ground-truth bounding-box regression targets *v = (v_x_, v_y_, v_w_, v_h_)*, where *x, y, w*, and *h* denote the two coordinates of the box center, width, and height, respectively. We used “FloodImageClassifier” Python package [13] to implement Fast R-CNN for image segmentation and classification. The results of Fast R-CNN were then fed into an object removal system to detect objects and reconstruct the image in a plausible manner by using exemplar-based inpainting method [1]. Exemplar-based inpainting algorithm uses a combination of texture synthesis and inpainting methods to identify the target region which needs to be filled in and a source region which is used as a reference to fill in the target regions. Final object removal pipeline takes an image as input, detects the location of various objects within the image and produces an image with the detected objects removed as the final output. This task is performed in order to detect the edges of the water surface using Canny Edge Detection [13] as it calculates the surface areas of water which in turn are used to determine the water level and severity. The severity and risk of flooding is identified based on aspect ratio presented in Table 1 [13]. The source code of “FloodImageClassifier” is available via GitHub [13].

**Table 1:** Water levels with associated aspect ratios and flood severity and risk estimation [13].

Water Level	Aspect Ratio	Flood Severity and Risk
Level 1	>1.8	Mild
Level 2	1.62 – 1.8	
Level 3	1.44 – 1.62	
Level 4	1.26 – 1.44	

Level 5	1.08 – 1.25	Moderate
Level 6	0.90 – 1.08	
Level 7	0.72 – 0.90	
Level 8	0.54 – 0.72	

Level 9	0.36 – 0.53	Severe
Level 10	0.18 – 0.36	
Level 11	<0.18	

### 3.2 Fast R-CNN Implementation

FloodIMG is utilized to train Fast R-CNN architecture for flood label detection. LabelImg was utilized to annotate FloodIMG photos with eight pre-defined item categories, including vehicle, person, forest, tree, traffic sign, residential area (i.e., houses), water vessels (i.e., boats, ships, etc.), and critical infrastructure (bridges, dams, etc.). Fast R-CNN predicts the class label, bounding box, and mask for the objects in an image. To perform Fast R-CNN, FloodIMG datasets were divided into training and testing sets at a ratio of 9:1, i.e., 90% for training and 10% for testing, along with their respective .xml files. As of >9200 images, 7,400 were used for training while >1,800 images were used for testing. Vehicle, person, forest, tree, traffic sign, residential area (i.e., houses), water vessels (i.e., boats, ships, etc.), and bridges/dams were among the object categories for which the prediction scores of Fast R-CNN was determined. The object categories are defined and coded in “FloodImageClassifier” Python package [10]. Once the detection results were generated, the package removes each of the detected objects and reconstructs the image by filling the void spaces using exemplar based inpainting (see Criminisi et al., 2003). Image inpainting model substituted convolutional layers with partial convolutions and masked the updates [13]. This algorithm successfully identified the target region which was filled using the surrounding areas of the target region as reference.

**Fig. 5:**
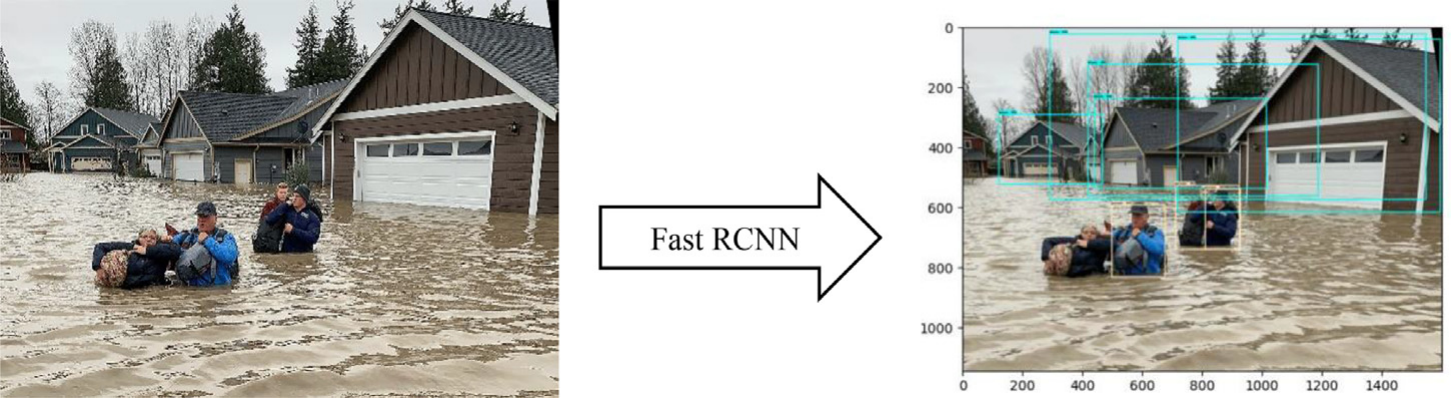
Fast R-CNN detection results. Input image (left) and detection results (right).

**Fig. 6:**
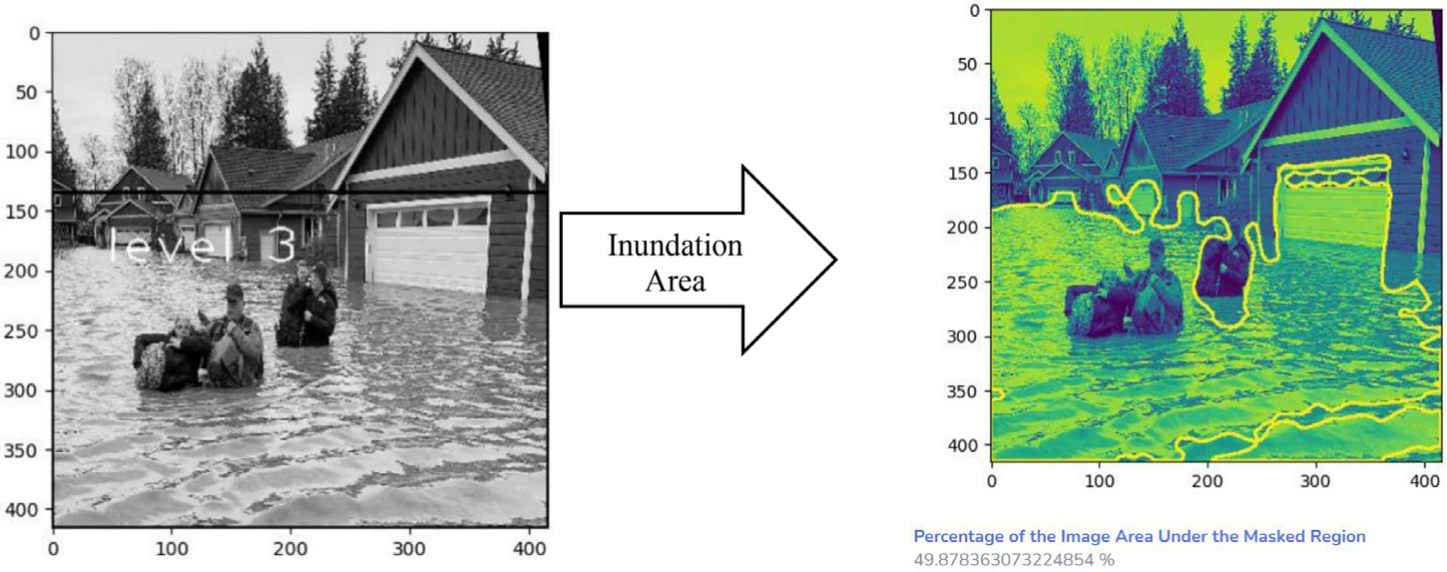
Flood severity level (left) and inundation area (right).

**Fig. 7:**
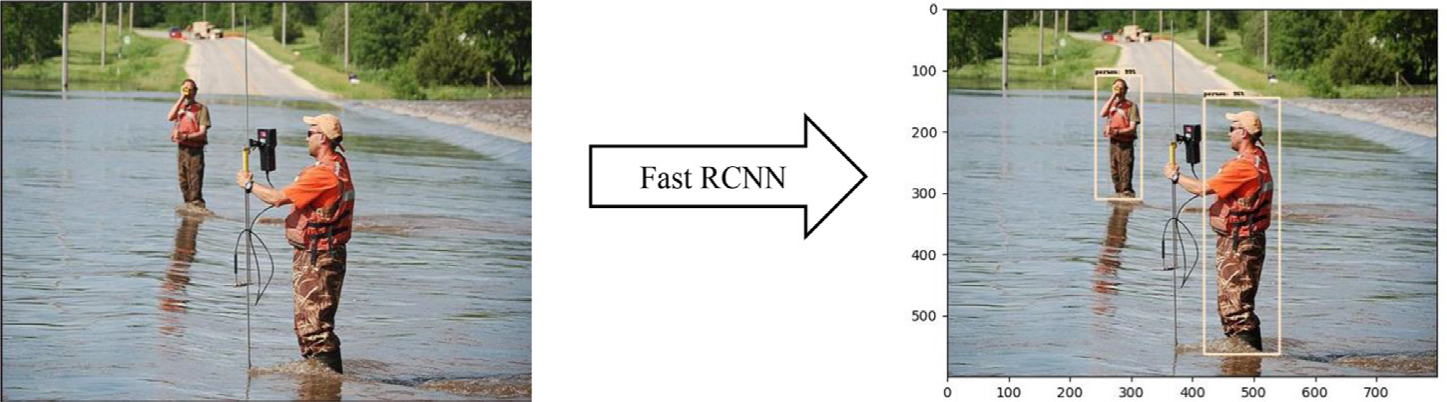
Fast R-CNN detection results. Input image (left) and detection results (right).

**Fig. 8:**
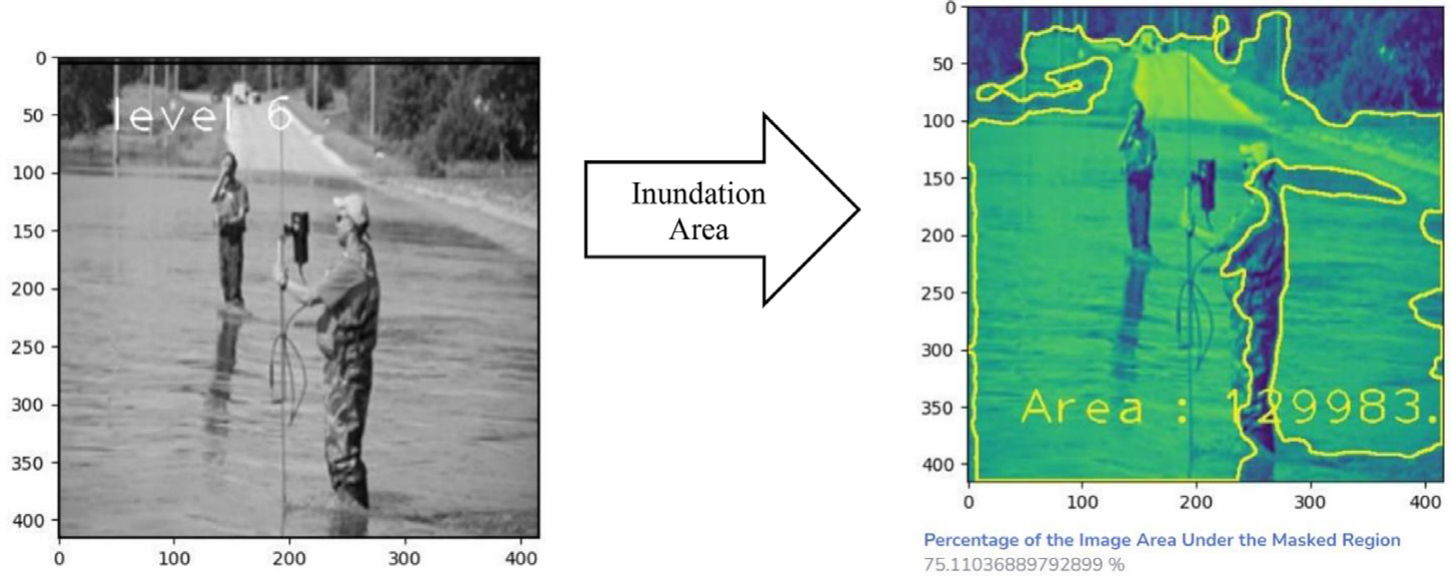
Flood severity level (left) and inundation area (right).

Figs. 5 and 7 depict the detection outcomes, while Tables 2 and 3 display the detection scores obtained by running Fast R-CNN model, on images present in Figs. 5 and 6, using FloodIMG dataset. As shown in Figs. 6 and 8, the edges of the water surface (i.e., draw the contours) was detected and then the area of the water surface was calculated. A bounding box was then drawn around the contour and its aspect ratio were calculated (i.e., the ratio between the width and the height of the water surface). We used the aspect ratio value to identify the water level class that was printed on the original image. Based on these classification, Figs. 6 and 8 showed respectively, floodwater levels 3 and 6, as well as 173056- and 11022-pixels areas. All these figs. identified mild to moderate flood risk and severity conditions based on Table 1 water levels. Fig. 6 showed around 50% of the area is flooded while Fig. 8 demonstrated 75%. Figs. 5 and 6 were taken from the test set of the data.

**Table 2:** Prediction scores of the image used in Fig. 5.

Object	House	House	House	House	House	House	Person	Person	Person	Classification Results	Flood Severity and Risk
Prediction score	97%	96%	84%	83%	58%	46%	83%	58%	45%	Flooding	Mild

**Table 3:** Prediction scores of the images used in Fig. 6.

Object	Person	Person	Classification results	Flood severity and risk
Prediction score	99%	40%	Flooding	Moderate

While the Fast R-CNN model was capable of detecting the above-mentioned object labels accurately, the model was unable to detect certain object labels such as trees, residential areas (i.e., houses) if the objects appearance were too small in the images. Fast RCNN algorithm has a number of combinations of convolutions followed by a pooling layer. While this allows the network to resize the images from ∼600×600 resolution down to ∼30×30, the small object features extracted on the first layers may disappear somewhere in the middle of the network and never actually count for detection and classification steps [13]. This is the reason why Fast R-CNN struggles with detecting small objects. In addition, errors and mismatches in the images such as image background, darkness in surface water, water reflection, and wavy surface water can cause erroneous or misdetection. While these types of errors are inevitable some time, we found that FloodIMG contains high quality images that can help provide accurate label detection and score calculation.

### 3.3 FloodIMG Application in Research and Future Direction

FloodIMG contains images from various flood events which would make the object detection for flood related research easy and straightforward. One usage of this dataset for rescue operations could be counting the affected number of objects during the flooding. Counting or detecting what objects are affected during the flooding helps authorities/citizens to understand what objects must be protected or removed from the possible flooding area.

An object detection model created with the image can also be used for rescue operations. The object detection model could be loaded to a CCTV camera that detects flooded objects which can help in rescue operations. This automatic detection of flooded objects helps to efficiently conduct rescue operations with the integration of CNNs. FloodIMG offers a public benchmark dataset for users who want to learn techniques and label detection methods on real-world data without much effort in data-preprocessing. We anticipate that FloodIMG's excellent image quality, variety of images, and labels make it a practical benchmark dataset for flood related image processing research. Because our dataset is readily available, it will enable the direct quantitative comparison between different deep learning approaches in a pragmatic manner. FloodIMG indeed enables training of neural network models that are performant in diverse scenarios, by lowering the cost of data capture and annotation required to excel in flood emergency research where ground truth data is scarce or hard to collect.

The authors anticipate that FloodIMG will enable the broader community of flood researchers to develop and train deep learning techniques for image-based flood monitoring and early warning system. Looking forward, we hope FloodIMG become a central resource for training of neural network algorithms and for a broad range of flood vision related research including image-based flood early warning system, road flooding alert, etc.

## Ethics Statements

The authors confirm that the provided data set and presented work strictly meet the ethics requirements for publication in Data in Brief as mentioned in https://www.elsevier.com/authors/journal-authors/policies-and-ethics. All data are fully anonymized and were collected and distributed under Twitter's Developer Policy 2021, DOT 2005, and GitHub [n.d.]. To collect Google data, we used the “Usage rights” Search tool (https://images.google.com/?gws_rd=ssl) to collect those images with redistribution rights.

## CRediT authorship contribution statement

**R. Karanjit:** Conceptualization, Software, Writing – original draft. **R. Pally:** Conceptualization, Methodology, Software, Data curation. **S. Samadi:** Conceptualization, Methodology, Writing – review & editing, Project administration, Funding acquisition.

## Declaration of Competing Interest

The authors declare that they have no known competing financial interests or personal relationships that could have appeared to influence the work reported in this paper.

## Data Availability

FloodIMG (Original data) (Kaggle).
